# Development of the Aquatic Competence Assessment for Children (ACA-C): A Tool for Measuring Personal Aquatic Competence Index

**DOI:** 10.3390/children12040484

**Published:** 2025-04-09

**Authors:** Rita Fonseca-Pinto, Juan Antonio Moreno Murcia

**Affiliations:** Sport Research Center, Miguel Hernández University, 03202 Elche, Spain; rita.fonseca@goumh.umh.es

**Keywords:** swim, children, measure assessment, validation, water safety

## Abstract

Background: This study developed and validated the Aquatic Competence Assessment for Children (ACA-C), an instrument designed to measure personal aquatic competence index in children aged 6 to 12 years. Aquatic competence is essential for water safety and the promotion of healthy habits, yet few validated tools integrate its multiple dimensions. The ACA-C is based on an ecological approach, considering the interaction between the child and various aquatic environments, both artificial (swimming pools) and natural (seas, rivers, and lakes). It is structured into three dimensions: aquatic literacy, drowning prevention, and environmental education, facilitating its application in diverse contexts. Methods: The ACA-C was validated using the Delphi method, with experts in aquatic education and safety. Kendall’s W coefficient of concordance was employed to measure the level of agreement among judges, ensuring rigorous criteria for item selection. Additionally, pilot studies were conducted with children in controlled aquatic environments to refine the instrument’s structure and content. Results: The results demonstrated high reliability and validity of the ACA-C for assessing children’s aquatic competence. This tool enables the identification of both actual and perceived competence levels, guiding pedagogical strategies for improvement. The inclusion of environmental education reinforces a comprehensive approach, fostering safety, responsible decision making, and enjoyment of aquatic environments. Conclusions: This study provides a validated instrument for use in educational and preventive contexts, contributing to water safety and well-being.

## 1. Introduction

The aquatic environment serves various recreational, educational, sports, and therapeutic purposes, depending on the context in which it is experienced. Whether in artificial settings such as swimming pools or natural environments like seas, rivers, and lakes, its influence on human development is undeniable. Understanding and developing aquatic competence is therefore essential to responding effectively to the demands of these environments.

Everyone should possess a minimum level of aquatic competence to safely engage with water, enjoy its benefits, and adopt appropriate behaviors. Navigating aquatic environments requires decision-making and emotional regulation skills, both individually and in groups. This raises a critical question: does perceived competence align with actual competence?

To help children make informed and safer decisions in aquatic settings, assessing their aquatic competence is essential. Such evaluation enhances the awareness of their true skill level across different scenarios and situations. While several scales exist to measure aquatic competence [1,2,3,4,5,6,7,8,9] many fail to capture its full scope, particularly in terms of cognitive and emotional responses to diverse aquatic challenges.

To address this gap, we developed an aquatic competence measurement scale based on an ecological perspective. This instrument provides an individualized assessment of children aged 6 to 12 years through a Personal Aquatic Competence Index (PACI), structured around three core dimensions: aquatic literacy, prevention, and environmental education. Designed for both artificial (pools) and natural (seas and rivers) aquatic environments, it facilitates comprehensive or partial evaluations, either individually or in groups. Additionally, it serves as a valuable pedagogical tool for designing aquatic education programs, promoting safety, awareness, and enjoyment of aquatic spaces.

### 1.1. Concept of Aquatic Competence and Decision Making

The concept of aquatic readiness, introduced by Langendorfer and Bruya (1995) [10], has been fundamental in shaping the current understanding of aquatic competence. Given the diversity of aquatic environments and practice modalities, individuals must develop the ability to adapt to different conditions [11]. Various settings, such as swimming pools, seas with changing currents and visibility, and rivers with diverse riverbeds, significantly influence human behavior in the water. Moreover, the aquatic environment carries inherent risks, with accidents ranging from minor negative experiences to fatal incidents. This is particularly relevant for children, as highlighted by Morrongiello et al. (2007) [12], who emphasize that children make critical decisions during play, including in aquatic activities, where emotional reactions play a key role in their behavior. Understanding these emotional responses is crucial to explaining why children choose to take or avoid risks in aquatic settings.

Taking into account sociocultural factors, individual characteristics, and social interactions, this study adopts the multidimensional approach to aquatic competence proposed by Fonseca-Pinto and Moreno-Murcia (2023) [13]. This integrative perspective views aquatic practice as a tool for human development, emphasizing the need to democratize access to aquatic activities, adapt to diverse aquatic contexts, and design educational programs tailored to learners’ needs.

**Figure 1:**
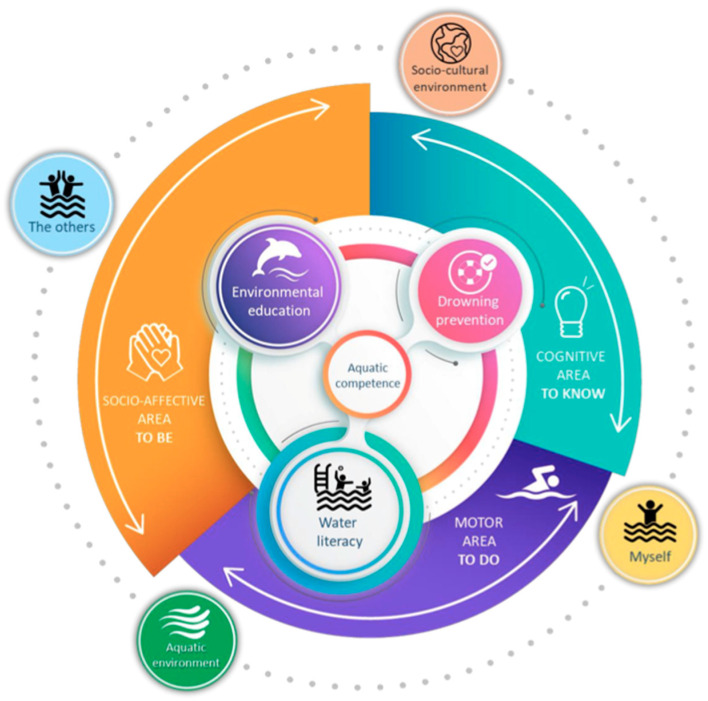
Multidimensional conceptualization of aquatic competence [13].

Decision making in this context involves three types of know-how (Figure 1): to be (which encompasses attitude and emotional regulation), to know (which refers to theoretical knowledge), and to do (which implies the ability to respond to environmental demands). From this ecological and innovative perspective, decision making is framed around a key guiding question: “Is this environment swimmable for me?”

### 1.2. Measuring Instruments for Aquatic Competence

The design of aquatic education programs must take into account their impact on health, skill development, and drowning prevention. In this context, measurement instruments play a fundamental role in assessing competencies, instructional strategies, and students’ specific needs.

Despite the variety of available tools for evaluating aquatic skills, the number of validated instruments remains limited. These tools can be classified into three main categories: instruments that measure aquatic competence [1,2,3,4,5,6]; those that assess perceived aquatic competence [14]; and tools that adopt a dual-perspective approach, evaluating both perceived and actual aquatic competence [15].

These instruments cater to different age groups, ranging from pre-schoolers [7,8,9] to school-aged children (6 to 14 years) and even young adults [16,17]. This diversity highlights the cross-cutting and cross-cultural nature of drowning prevention and aquatic competence as a global concern.

Most studies focus on assessments conducted in swimming pools, despite the fact that a significant proportion of drownings occur in natural environments, often under unexpected conditions, such as without proper swimwear or safety measures. Moreover, many existing assessments fail to holistically evaluate aquatic competence across multiple dimensions—motor, cognitive, and perceived competence.

Beyond assessing aquatic competence, this study emphasizes the role of the family as a crucial element in drowning prevention, health promotion, and overall well-being, as highlighted by the World Health Organization [18]. Families play a pivotal role in encouraging children’s engagement in aquatic activities as part of a healthy lifestyle [3]. When perceived competence aligns with actual aquatic competence, children’s decision making in aquatic settings becomes more accurate and contextually appropriate. However, from an ecological perspective, there remains uncertainty about how children respond in real-life situations, where environmental conditions and stress factors may differ from controlled assessments.

These measurement instruments also hold significant pedagogical value, as they help identify learning gaps, analyze behavioral patterns, and refine educational content to ensure the selection of appropriate teaching strategies for both individuals and groups.

From a scientific perspective, these tools are essential in supporting evidence-based practice, moving beyond subjective opinions or traditional approaches. By providing objective data, evaluation tools serve as references for personal development, learning, and long-term safety in aquatic environments [3]. Additionally, they respect individual learning paces and needs, ultimately fostering a more effective, student-centered teaching process.

### 1.3. The Current Study

**Figure 2:**
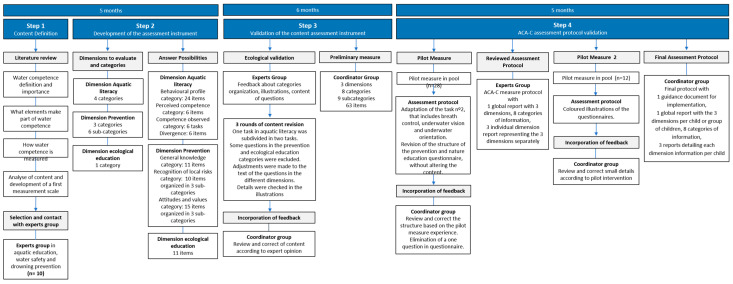
Timeline of the implementation phases of the development of the ACA-C assessment tool.

This study aimed to develop and validate the ACA-C, a scale designed to measure the PACI in children aged 6 to 12 years. To ensure the validity and reliability of the instrument, a systematic process was undertaken. First, content validity was assessed through expert judgment using the Delphi method. Next, the feasibility and comprehensibility of the instrument were analyzed qualitatively to evaluate its clarity. Additionally, the internal structure and psychometric properties were examined to establish the scale’s reliability. Finally, the relevance of each item was verified concerning the identified dimensions, ensuring its applicability in assessing aquatic competence effectively. The validation of the ACA-C followed a structured process consisting of four main stages (Figure 2).

## 2. Methods

### 2.1. Participants

The coordinating group consisted of two experts in aquatic activities: one with extensive experience in teaching aquatic education across different age groups and another specialized in teaching and research in this field. Both possessed a strong understanding of the Delphi method and maintained effective communication. Their main responsibilities included the following: (a) conducting a literature review on methods for assessing aquatic competence; (b) developing a proposed scale to measure aquatic competence; (c) Selecting and contacting expert consultants; (d) Organizing and managing expert feedback, and striving for consensus to validate the content of the scale.

To ensure content validity, a purposive sample of 15 experts was selected, of whom 10 agreed to participate and 5 were unavailable. The participants were from Portugal, Brazil, Spain, and Uruguay, all with extensive experience in aquatic education, teaching, research in aquatic activities, as well as safety and drowning prevention. Among them, five had university teaching experience.

The authors emphasize that the results obtained from the consultation were the responsibility of the expert group, who contributed at every stage of the scale development process, including mathematical calculations. Additionally, they ensured that the content was not disclosed among the expert consultants, maintaining confidentiality throughout the process.

### 2.2. Procedures

This study was approved by the Ethics Committee for Research at Miguel Hernández University of Elche with the code DCD.JMM.01.22. The Delphi method was used with a panel of experts with extensive experience in the fields of research, teaching in aquatic activities, and drowning prevention. The process included several stages, outlined in the timeline, to determine content validity through expert judgment (Figure 2).

**Figure 3:**
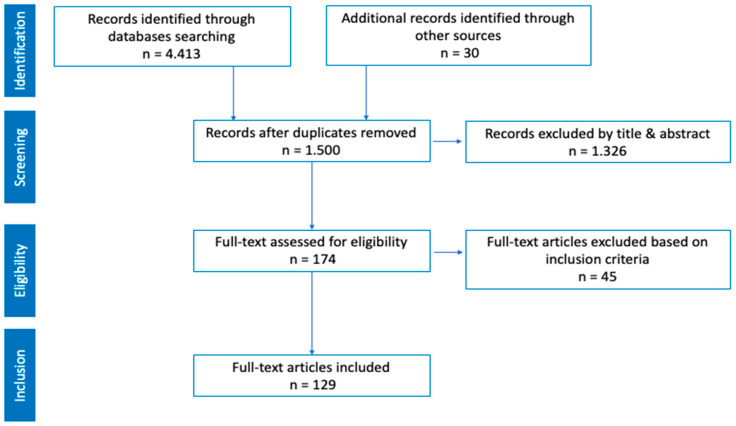
PRISMA diagram of the literature review on aquatic competence.

**Figure 4:**
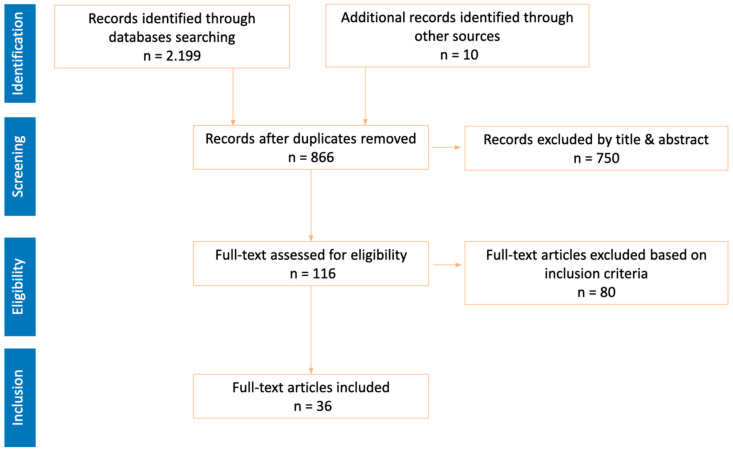
PRISMA diagram of the literature review on drowning prevention.

**Figure 5:**
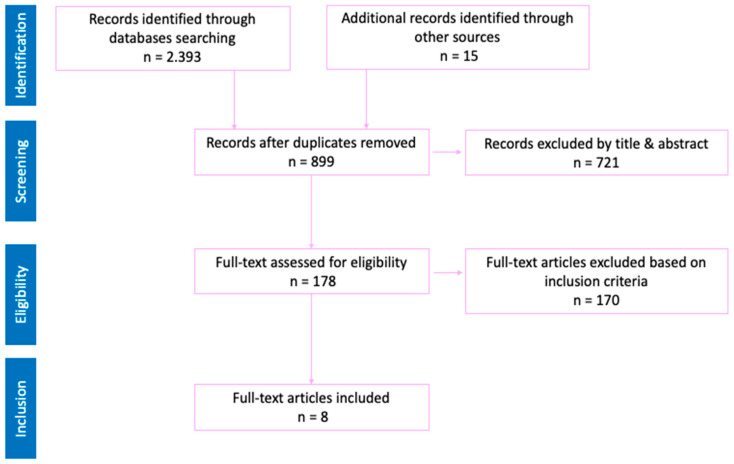
PRISMA diagram of the literature review on measuring connection with nature.

Step 1. Definition of the assessment instrument construct. The content construction was based on an extensive literature review, focusing on three main axes: the concept of aquatic competence (Figure 3), its core components (Figure 4 and Figure 5), and the existing measurement scales. Based on this review, the coordinating group carried out a process of discussion and analysis, resulting in the first version of the measurement protocol. This protocol was organized into three dimensions (aquatic literacy, drowning prevention, and environmental education) and eight complementary areas (personal identification data, behavioral profile, discrepancy between perceived and observed competence, aquatic literacy, prevention, environmental education, PACI, and practical recommendations). This structure facilitates comprehensive characterization at both the individual and group levels.

The coordinating group conducted a bibliographic search on the concept of aquatic competence, its social value [19], and methods of assessing aquatic competence at the individual and group levels [1,2,3,4,5,6,7,14,15]. Additionally, studies on measuring the connection with nature were reviewed [20,21,22,23]. To ensure scientific rigor, databases such as Web of Science, Scopus, Science Direct, and Sport Discus were consulted. Some of the terms used in the search were water competence, aquatic competence, aquatic skills, swim skills, drowning prevention, water safety, measure, assessment tool, connection with nature, relationship with nature, and children.

The age of previous sources was a key eligibility criterion, along with the scientific validity of the measurement scales analyzed. Both factors were decisive in the exclusion of multiple previously published documents, primarily due to limitations in their validity.

Given the innovative aspect of certain contents, such as the connection with nature, a previously validated scale for terrestrial environments was adapted to the aquatic context. Drawing from a vast information base and seeking to embrace an ecological and nonlinear view of achievements in aquatic competence, the authors first established a global perspective on the concept of Fonseca-Pinto and Moreno-Murcia (2023) [13].

For the collection of information, and the design and validation of the instrument, the Delphi method [24] was applied, based on the consultation of a panel of experts. Fifteen experts were selected through purposive sampling, based on the following criteria: (1) specific experience in teaching aquatic activities; (2) scientific background and number of publications in the field of aquatic competence; (3) expertise in the dimensions being assessed (aquatic literacy, drowning prevention, or environmental education), and (4) availability and ethical commitment to actively participate in all rounds of the Delphi process.

In this way, we ensured an expert, diverse, and relevant participation, which contributed conceptual and technical robustness to the instrument’s validation process.

The experts, from different nationalities (Portugal, Brazil, Spain, Uruguay), received an e-mail invitation to participate in the study. Together with the invitation, the experts were sent a cover letter describing the research and the global conceptualization of aquatic competence according to [13]. However, five experts declined the invitation due to the impossibility to guarantee impartiality or because of possible conflicts of interest, resulting in a final participation of 10 experts.

Step 2. Development of the assessment instrument. This second stage was structured in three phases: (a) answering the question to be measured and justifying its relevance, (b) defining the dimensions and assessment categories, and (c) exploring the response options for the initial version of the measurement scale. The responsibility for this process was with the coordinating group.

Step 3. Assessment instrument validation. The organization of this phase was structured in two parts: (a) ecological validation and (b) preliminary version of the scale.

Ecological validation. For the validation of the instrument, the guidelines of Escobar-Pérez and Cuervo-Martínez (2008) [25] were followed, establishing a clear objective for the expert judgment to validate the content of the instrument designed by the coordinating group. The instrument was structured in three dimensions, each with its respective categories and measurement items.

Given the specificity of the content and the profile of the participating experts, two groups were formed in the first phase. Group A was in charge of validating the aquatic literacy dimension and had five experts (1 man and 4 women), while group B evaluated the prevention and environmental education dimension, with the participation of five experts (1 woman and 4 men). Both groups analyzed the content in terms of clarity, coherence and relevance. Once this phase was completed, all experts were asked for a comprehensive validation of the instrument, considering its relevance, sufficiency and adequacy.

Following the methodological recommendations of Escobar-Pérez and Cuervo-Martínez (2008) [25], several documents essential to the process were provided to the experts. These included an explanation of the importance of the evaluation of measurement instruments in the research, the theoretical framework of each dimension and its relevance, and detailed instructions for the completion of the instrument, together with the evaluation criteria. For the environmental education and prevention dimension, clarity, relevance, coherence and consistency were assessed, while for the aquatic literacy dimension, task ecology and transfer of competences were considered. Additionally, an evaluation record sheet and the initial version of the scale were provided.

The documents were sent via email, facilitating communication between the coordinating group and the experts. The experts rated the indicators on a scale from 1 to 4 (where 1 represents a criterion not met and 4 indicates a high level of compliance) and provided qualitative observations that helped to obtain a text that was easier to understand for the target population, relevance of the context, terms used, order of the questions and details of the drawings. A maximum period of 30 days was established for completing the evaluation.

Once the review period ended, the coordinating group gathered and analyzed the experts’ feedback, discussed the recommendations, and made the necessary adjustments to the experimental version of the instrument. Subsequently, the changes were shared again with the experts for validation. Due to the complexity and novelty of the evaluated content, this process was repeated three times for the aquatic literacy dimension and four times for the prevention and environmental education dimensions until the final version of the instrument was achieved.

Preliminary version of the instrument. Once the content validation for each of the three dimensions was agreed upon separately, the process moved forward to the overall evaluation of the scale. For this purpose, a group of four experts was selected to analyze the indicators of clarity, relevance, and coherence.

Finally, after this review process, a consensus was reached among the experts. Based on all the collected information, a pilot study was designed, for which a guiding document was developed, titled Implementation Protocol of the ACA-C.

Step 4. Pilot study. In this final phase, we conducted a pilot study to test and verify the procedures in a practical context. To this end, we developed an explanatory document titled Implementation Protocol of the ACA-C.

To ensure a proper understanding of the protocol and the responsibilities during the measurement process, we organized two meetings with the evaluation team—one in person and another online. Additionally, to invite families to participate, and with the support of the swim pool team’s, we held two online informational sessions for parents. During these sessions, we presented the implementation protocol, explained the study’s objective, emphasized the requirement to sign the informed consent form, and addressed any questions.

In the first pilot implementation, 28 volunteer children participated, ranging in age from 6 to 12 years (13 boys and 15 girls). The measurement was conducted in an indoor swimming pool measuring 25 m × 12.5 m, with a variable depth from 1.20 m to 2 m and a water temperature of 29 °C.

The participants were organized into two age groups (6–8 years and 9–12 years), with a one-hour interval between the start of each measurement. Group A began with the practical assessment before completing the questionnaires, while Group B followed the reverse order. Within each group, the children were further divided into small subgroups to rotate through different task stations, with continuous swimming being the only activity conducted collectively for space management reasons.

The team of evaluators consisted of 10 people, including two people responsible for the preparation and layout of the tasks, one of whom supervised and supported the entire process. Additionally, one evaluator was in charge of the photographic documentation, while the rest of the team was distributed individually or in pairs for data collection.

Following this first implementation, the need for some adjustments was identified, leading to a second pilot test one year later, in a different facility and with a different group of children. In this case, the indoor pool had dimensions of 25 m × 17 m, a variable depth between 1.20 m and 1.60 m, and a water temperature of 28 °C. Twelve children (6 boys and 6 girls) aged between 6 and 12 years took part, under the supervision of a team of six evaluators. On this occasion, one leader was responsible for the preparation, set-up, supervision and general support in all tasks, while the remaining five evaluators were distributed among the different activities, recording the corresponding data. Some tasks were assessed by the same person, such as safe entry and underwater swimming, as well as lifejacket use and safe exit.

In both implementations, parents or legal guardians signed informed consent prior to children’s participation. The assessment was completely voluntary, allowing any child to refuse or drop out of the evaluation at any time without consequences or comments.

### 2.3. Data Analysis

Qualitative data were analyzed using content analysis. For the data obtained from the expert panel’s responses, it was necessary to establish a consensus criterion beforehand for item validation. There is no single definition of consensus, but one of the most commonly used approaches is the percentage of agreement [26].

According to Humphrey-Murto et al. (2016) [27], a 70% consensus among experts who selected ’agree’ or ’strongly agree’ for an item was considered sufficient for its acceptance. Conversely, a 70% response rate of ’disagree’ or ’strongly disagree’ led to its elimination, while items with intermediate scores were reassessed in the next evaluation round.

During the different phases of the Delphi method (validation of the construct for each dimension and, subsequently, the overall instrument), experts were provided with a document containing the instrument and evaluation criteria tailored to each section’s characteristics. In all cases, assessments were conducted using a Likert scale ranging from 1 (does not meet the criterion) to 4 (high level of compliance).

In the aquatic literacy dimension, experts evaluated the following components: profile questions, task illustration, task description, and evaluation criteria. The assessment was conducted based on the following categories: (a) clarity of profile questions’ content, analyzing syntactic and semantic adequacy; (b) relevance of the task, verifying whether the activity assessed the described competence; (c) clarity of the task content, ensuring the explanation was easily understandable with appropriate syntax and semantics; (d) ecological validity of the task, considering whether the activity involved a certain degree of injury risk to activate emotional responses; (e) competence transferability, determining whether the task facilitated the application of competence in other aquatic environments; (f) feasibility, ensuring that the task was suitable for the developmental stage of the target population; and (g) relevance, assessing whether the task was essential or important for measuring aquatic competence.

In the prevention and environmental education dimension, organized in questionnaire format, the following evaluation criteria were applied: (a) sufficiency, determining whether the questions were adequate to measure the intended competence; (b) clarity of question content, verifying comprehensibility at a syntactic and semantic level; (c) coherence, assessing the logical relationship between the question and the dimension being measured; and (d) relevance, determining whether the question was essential or important within the instrument. Since some questions included illustrations, an additional criterion, congruence, was introduced to verify whether the image accurately represented the proposed description.

The overall protocol of the instrument was also evaluated by experts according to the following aspects: (a) clarity of explanation, determining whether the description of the evaluation objectives was clear; (b) relevance, analyzing whether the proposed parameter was essential for determining the PACI; (c) strategy, assessing whether the method used to determine PACI was appropriate; and (d) coherence, ensuring that descriptors were representative of the information necessary to characterize the student.

To determine the degree of agreement among experts regarding the final proposal, Kendall’s W coefficient of concordance was used, considering both its numerical value and statistical significance. This coefficient ranges from 0 to 1, where values closer to 1 indicate a higher level of agreement among evaluators. According to Schmidt’s (1997) [28] classification, the results were interpreted as follows: W ≈ 0.1 indicates very weak agreement, W ≈ 0.3 represents weak agreement, W ≈ 0.5 corresponds to moderate agreement, W ≈ 0.7 suggests high agreement, and W ≈ 0.9 indicates an exceptionally high agreement. In addition to the W value, the *p*-value was considered to determine the statistical significance of the observed agreement, with a reference threshold of *p* ≤ 0.05. A *p*-value equal to or below this threshold indicated that the concordance among evaluators was not due to chance. Therefore, in interpreting Kendall’s W coefficient, both its magnitude and corresponding *p*-value were taken into account, ensuring a rigorous and objective assessment of the degree of consensus among experts.

## 3. Results

### 3.1. Step 1: Definition of the Assessment Instrument Construct

A comprehensive review of the main publications on aquatic competence was conducted, highlighting the work of Stallman et al. (2017) [19], which identifies 15 key aquatic competencies essential for drowning prevention and provides pedagogical recommendations for their development. The relevance of these competencies has been widely recognized in the scientific literature, as referenced in studies such as Button et al. (2020, 2017) [29,30], Drowning Prevention Auckland (2020) [31], Guignard et al. (2020) [11], Karatrantou et al. (2019) [32], Moreno-Murcia et al. (2020) [4], Moreno-Murcia and Ruiz (2019) [33], Ortiz et al. (2021) [34], Peden et al. (2017) [35], and Willcox-Pidgeon et al. (2020) [36]. These works emphasize that aquatic competence is crucial not only for drowning prevention but also for its role in recreational, sports, educational, and therapeutic contexts, both in natural and artificial aquatic environments.

Beyond its preventive function, aquatic competence is recognized as a key component of physical literacy [37,38,39]. Its development promotes an active lifestyle and strengthens the connection with nature through outdoor physical activities [40]. Various studies have documented the benefits of this interaction [41,42], highlighting the transferability of skills from aquatic to natural environments [11].

Existing measurement scales for evaluating aquatic competence have primarily focused on observing motor skills and drowning prevention through questionnaires [1,2,3,4,5,15]. However, no studies have been identified that comprehensively address all aquatic competencies. Additionally, researchers have recognized the need to broaden the approach to child development and decision making [12,43], considering the inherent risks associated with children’s interactions with water in various contexts and conditions [11,19,30,34].

Ensuring ecological validity in the measurement scale is essential. This involves designing assessment scenarios that physically challenge the child and elicit emotional responses while maintaining safety and well-being, both physically and emotionally. Based on the literature review, three fundamental dimensions were defined for measuring the PACI: (a) aquatic literacy, (b) drowning prevention, and (c) environmental education.

### 3.2. Step 2: Development of the Assessment Instrument

Building on the previous review, the coordinating group designed an inventory of assessable competencies within the dimensions of aquatic literacy and prevention, ensuring the inclusion of the 15 aquatic competencies identified in the literature. To expand the instrument’s scope, a third dimension focused on environmental education was added, introducing an innovative approach to the measurement scale.

Once the dimensions were established, the coordinating group structured the document into dimensions, categories, and subcategories, drafting all items in alignment with the theoretical framework of aquatic competence. The initial version of the instrument included six tasks with a total of 79 items, distributed as follows: 24 items for aquatic literacy, 45 items for prevention, and 10 items for environmental education.

### 3.3. Step 3: Validation of the Assessment Instrument

The validation process revealed the necessity of an iterative approach to reaching expert consensus. While the aquatic literacy and environmental education dimensions required three rounds of consultation, the prevention dimension required four rounds before agreement was achieved. However, the overall structure of the scale gained consensus and acceptance from the first round.

Significant modifications were made to the instrument during validation. In the aquatic literacy dimension, an additional task was incorporated to fully meet the assessment objectives. Across all dimensions, redundant or non-consensual questions were removed to improve efficiency and clarity.

Statistical results indicated an appropriate Kendall’s coefficient above 0.72 was obtained, which, according to Schmidt’s (1997) [28] classification, indicates a high level of agreement among the evaluators. Furthermore, the analysis was statistically significant (*p* < 0.01), reinforcing the reliability of the validation process. Qualitative feedback was also carefully considered, particularly in wording refinements. The coordinating group was responsible for implementing necessary adjustments throughout the validation process, ensuring consistency and coherence in the final instrument.

### 3.4. Step 4: Pilot Study

The first pilot implementation of the assessment protocol identified key adjustments needed, particularly in Task 2, which assessed breath control, underwater swimming, and underwater vision. This task posed implementation challenges in various aquatic environments due to depth variations, currents in natural settings, and safety concerns. Additionally, the need to simplify question wording for young participants and improve the document structure for greater engagement and clarity was recognized.

Following the second measurement, further refinements were implemented. All illustrations in the questionnaires were colored, and decision-making questions preceding the tasks were visually enhanced to increase participant engagement. Minor adjustments were also made to the protocol document to improve its clarity and effectiveness. Task 2 was redesigned to better align with the measurement objectives, ensuring feasibility and relevance.

As a result of these iterations, the final version of the ACA-C (in Supplementary Material) was structured as follows (see Annex): an explanatory document detailing the assessment protocol, which includes seven tasks and a total of 112 items distributed across the three dimensions. The aquatic literacy dimension consists of 69 items, the prevention dimension of 36 items, and the environmental education dimension of 7 items. This comprehensive set of items provides sufficient data to characterize the PACI in children aged 6–12 years. Additionally, the protocol facilitates flexible application, making it a valuable tool for both educational and research contexts in assessing and developing aquatic competencies.

## 4. Discussion

Aquatic competence and its assessment are crucial at all stages of life. However, there is a significant lack of scientifically validated instruments capable of accurately characterizing aquatic competence in diverse settings while also offering pedagogical recommendations for its development. Addressing this gap, the present study aimed to design and validate an instrument to measure the PACI, ensuring its applicability in educational and practical contexts.

As a result, this study introduces the ACA-C, an optimized tool that enables aquatic educators to assess PACI in children aged 6 to 12 years, either comprehensively or in specific dimensions. The thoroughness of the measurement scale is directly linked to the validity of its content and its ability to accurately describe the child’s behavioral profile, facilitating the personalization of pedagogical strategies.

This research details the construction and development of a self-report scale specifically designed to measure PACI. The tool provides valuable insights into individual characteristics and areas requiring further development, benefiting not only the child but also their family and aquatic educators. The scale’s design was grounded in an extensive literature review, broadening its focus to incorporate relevant social and ecological aspects, both present and future. The innovation of this instrument lies in its multidimensional approach to aquatic competence, extending beyond drowning prevention—which is viewed as a natural consequence of high competence levels fostered by a diverse, intentional, and structured pedagogical process.

The Delphi methodology was employed to develop the instrument, integrating qualitative and quantitative approaches to establish content validity. The validation process involved expert judgment, with items retained only if they achieved a significance level of 0.05 or higher, ensuring strong agreement among evaluations. Items that failed to reach consensus or relevance were systematically removed after multiple rounds of revision.

The preliminary version of the PACI measurement instrument comprises three dimensions, seven categories, three subcategories, and 85 items. This scale facilitates comprehensive assessment, yielding valid, detailed personal characterizations, while also offering the flexibility to conduct partial evaluations based on specific needs. Furthermore, it provides personalized recommendations beneficial for aquatic educators, families, and participants, with the overarching goal of enhancing aquatic competence.

Given the dynamic, variable, and unpredictable nature of aquatic environments, it is essential to view PACI as an initial reference point rather than an absolute measure. The interpretation of results must always be contextualized, considering both the specific environmental conditions and the individual characteristics of the child when interacting with water.

### Practical Implications

The PACI is a powerful tool that facilitates a comprehensive characterization of individuals by aligning their perceived aquatic competence with their actual abilities. This approach actively involves both the learner and their family, fostering a more effective teaching–learning process. Unlike traditional assessments that focus solely on technical skills in controlled environments like swimming pools, PACI adopts an innovative, complex, and multidimensional perspective, addressing not only physical actions but also cognitive and socio-emotional factors.

The cognitive, motor, and socio-affective dimensions of the PACI are inherently interconnected, making it impossible to isolate any one component without impacting the others. While different aspects of aquatic competence may be emphasized at various stages of learning, achieving high competence levels requires a holistic perspective that considers all dimensions collectively. This integrated approach ensures that skill development extends beyond technical proficiency, fostering a more well-rounded and adaptive aquatic competence in children.

## 5. Conclusions

The Aquatic Competence Assessment for Children (ACA-C) stands out for its integrative approach, combining preventive and protective aquatic competences [19] with an ecological pedagogical perspective that accounts for the diversity of aquatic environments. It also incorporates the influence of emotional and cognitive reactions on decision making in aquatic settings [44]. To assess the environmental education dimension within aquatic contexts, this study adapted the Nature Connection Scale of Ferrero et al. (2021) [22], focusing on attitudes and preferences toward activities in natural environments, addressing the lack of specific instruments in this area.

This research represents a significant advancement in the comprehensive assessment of aquatic skills during childhood. While the ACA-C has not yet been validated for children with disabilities or tested in natural aquatic environments, it aims to contribute to aquatic education and child development by promoting safety and enjoyment in aquatic settings. Rather than opposing the traditional concept of “knowing how to swim”, the ACA-C expands and redefines it. Before participating in any aquatic activity, it is essential for individuals to develop a strong PACI, which involves observing, interpreting, self-regulating emotions, making appropriate decisions, and identifying risks and assistance strategies when necessary. This holistic perspective ensures a positive and lasting experience in aquatic environments.

Beyond being an assessment tool, the ACA-C provides a valuable resource for designing personalized educational programs. It also facilitates the development of self-concept and personal beliefs through continuous evaluation, bridging the gap between subjective perception and actual competence.

Further research is needed to validate the ACA-C in natural environments and adapt it for children with disabilities. Additionally, it would be beneficial to explore the effects of different pedagogical methodologies on the PACI. These advancements could significantly enhance the tool’s practical application in swimming pools and educational settings, ultimately fostering a more active, safety-conscious, and well-informed society through effective aquatic education.

## Data Availability

Fonseca Pinto, R., & Moreno Murcia, J. A. (2025). Aquatic Competence Assessment for Children. Zenodo. https://doi.org/10.5281/zenodo.14999173 (accessed on 10 March 2025). The original data presented in the study are openly available in Workflow (instrument to measure aquatic competence index in children). Data are available under the terms of the Creative Commons Attribution 4.0 International and Creative Commons Attribution Share Alike 4.0 International.

## Supplementary Materials

The following supporting information can be downloaded at: https://www.mdpi.com/article/10.3390/children12040484/s1, Aquatic Competence Assessment for Children.
