# Supplementary material for: Development of the Aquatic Competence Assessment for Children (ACA-C): A Tool for Measuring Personal Aquatic Competence Index
Source: Children (Basel). 2025 Apr 9;12(4):484. doi: 10.3390/children12040484 (PMC12025921; doi:10.3390/children12040484)
Supplement: Supplementary file 1 [file children-12-00484-s001.zip › children-3562548-supplementary.pdf]

ACA-C

Aquatic Competence Assessment

FOR CHILDRENS

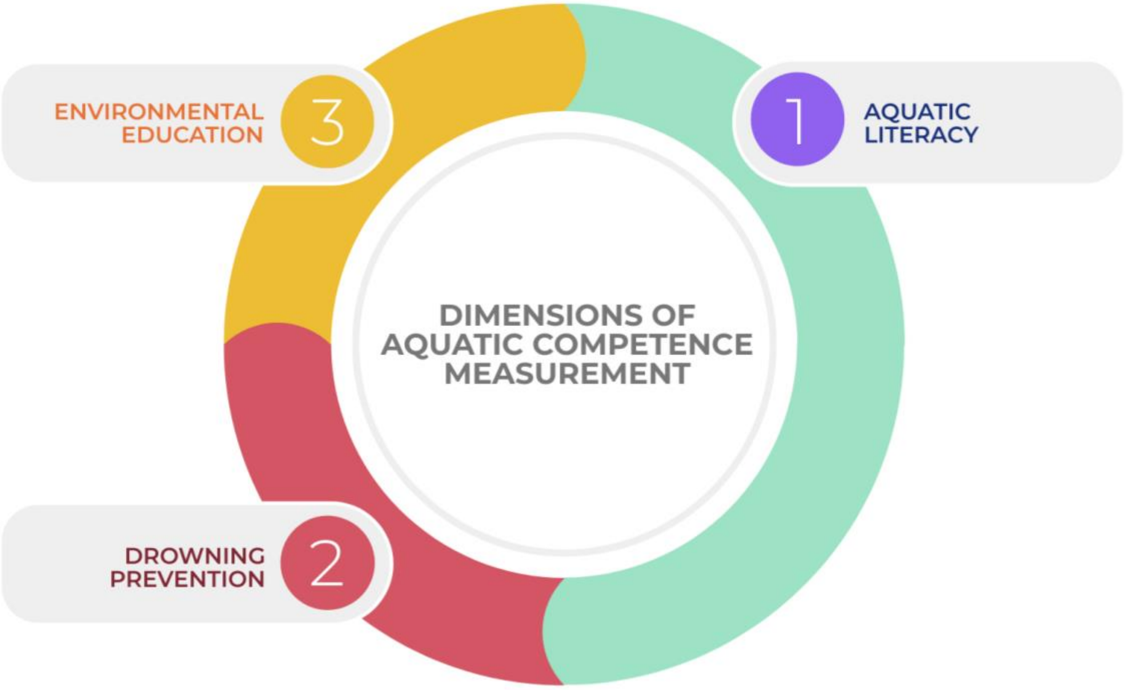
ENVIRONMENTAL EDUCATION
3
1
AQUATIC LITERACY
DIMENSIONS OF AQUATIC COMPETENCE MEASUREMENT
DROWNING PREVENTION
2

1.
MEASURING AQUATIC LITERACY DIMENSIÓN

Task 1. Measurement of ‘safe entry’ competence.

**Questions**	**1**	**2**	**3**
Do you think you could get hurt or drown if you enter the water in a deep area and come back to the surface alone?	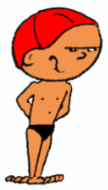 It doesn't hurt / I can't drown	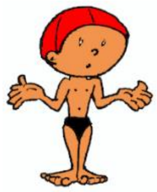 It can hurt me/can drown me	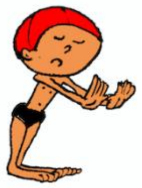 I can almost certainly get hurt/drown.
Do you think it is dangerous to enter the water in a deep area and come back to the surface alone?	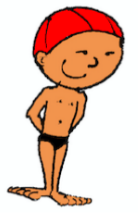 Nothing dangerous	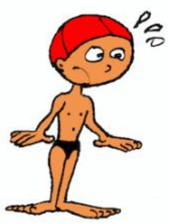 Danger	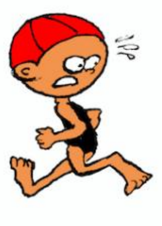 Very dangerous
Would you like to enter the water now in this deep area and come back to the surface alone?	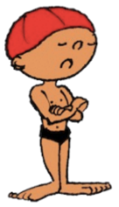 I wouldn't like to	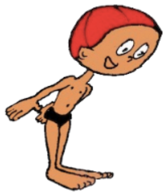 I would like to	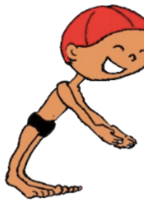 I would really like to
Are you afraid to enter the water now in this deep area and come back to the surface alone?	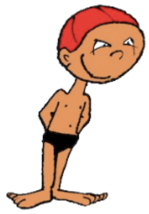 I don't feel afraid	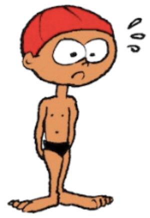 I feel afraid	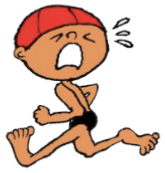 I feel very scared

Motor task
Entering into the deep water with autonomy.

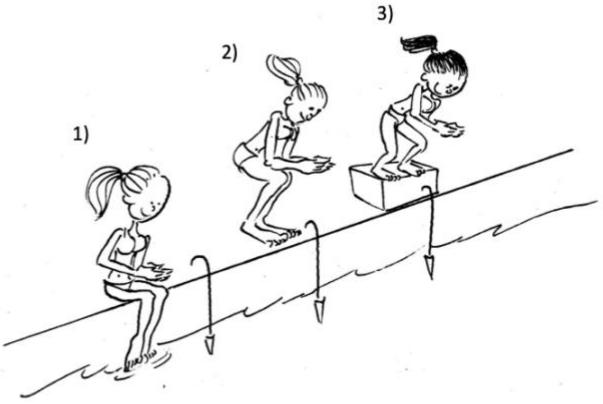

Selected task level	Evaluation criteria				
**What level of more complex task do you think you are able to do very well and want to do?**	**How the task is done**	**0** Decline to do the task.	**1** Make with help (with or without goggles)	**2** With goggles	**3** Without goggles
1) From a sitting position next to the water level (example: edge of the pool).<br>2) From a standing position aligned with the water.<br>3) From a height of about 50 cm from the water surface, from a standing position (e.g. starting block).	**Water entry**		**1** Entry into the water without submerging the head or entry into the water with help	**2** Entry with autonomy, submerging the whole body with discomfort (cleaning the face at the exit of the water, with closed eyes).	**3** Entry with autonomy (dive and return to the surface with controlled breathing, without cleaning the face and with eyes open).
**Time of execution:**	……....minutes………..seconds				

Task 2. Measurement of underwater competence and respiratory control.

Questions	1	2	3
Do you think you could get hurt or drown if you swim, without goggles, underwater, a U-shaped distance, through a hoop, pick up an object at depth and resurface under the mat?	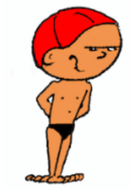 It doesn't hurt / I can't drown	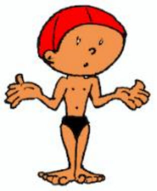 It can hurt me/can drown me	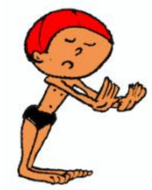 I can almost certainly get hurt/drown.
Do you think it is dangerous to swim, without goggles, underwater, a U-shaped distance, through a hoop, pick up an object at depth and resurface under the mat?	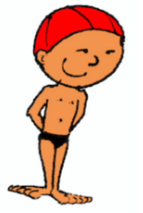 Nothing dangerous	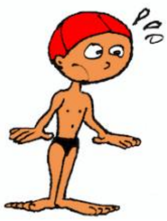 Danger	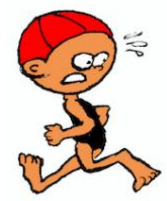 Very dangerous
Would you like to swim now, without goggles, underwater, a U-shaped distance, through a hoop, pick up an object at depth and resurface under the mat?	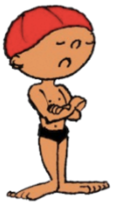 I wouldn't like to	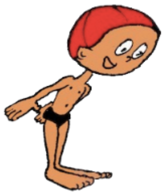 I would like to	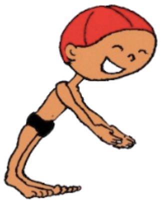 I would really like to
Are you afraid to swim now, without goggles, underwater, a U-shaped distance, through a hoop, pick up an object at depth and resurface under the mat?	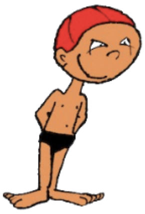 I don't feel fear	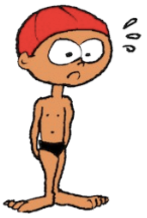 I feel fear	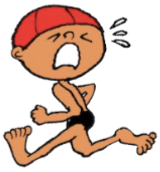 I feel very scared

Motor task

Complete an underwater circuit (fully submerged body) in a U-shape from the starting point to the finishing point. It is necessary to invert the position of the body, move with the body totally submerged inside a hoop, pick up an object at the bottom and return to the starting point under a mat.

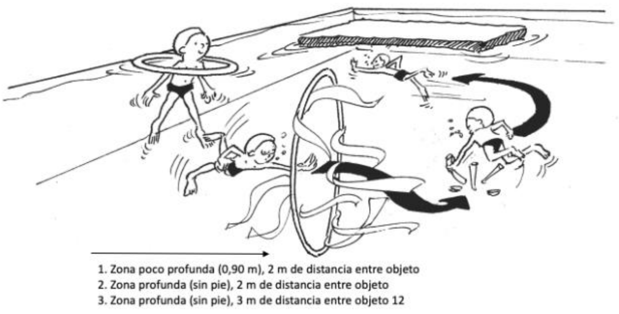

**Selected task level**		**Evaluation criteria**			
**What level of more complex task do you think you are able to do very well and want to do?**<br>1) Make the circuit in an area where you can touch the bottom (shallow).<br>2) Make it in an area where you can´t touch the bottom (deep).<br>3) Make it in an area where you can't touch the bottom (deep) and with the objects further away from each other.	**How the task is done**	**0**<br>Decline to do the task	**1**<br>Make with help (with or without goggles)	**2**<br>With goggles	**3**<br>No goggles
	**Reversal of body position** (feet higher than the head)		**1**<br>Cannot reverse the position of the body or submerge it	**2**<br>Reverses the body position and makes the immersion with assistance	**3**<br>Inverts the position of the body and immerses autonomously.
	**Underwater swim**		**1**<br>Need to hold the hoop and/or the mat to transpose it.	**2**<br>Makes the underwater circuit with the body close to the surface touching the material (hoop and/or mat).	**3**<br>Makes the underwater circuit with the body submerged without touching the material.
	**Breath control**		**1**<br>Breathing pause before each challenge (hoop, object and mat)	**2**<br>Pause to breathe somewhere along the circuit (hoop, or mat) or to stop to pick up the object.	**3**<br>Performs the entire circuit without breathing breaks
	**Underwater vision**		**1**<br>Fails to pick up the object until the 4th try	**2**<br>Pick up the object on the second attempt	**3**<br>Collect the object on the first try
**Time of execution:**	……….minutes………..seconds				

Task 3. Measurement of propulsive competence.

Questions	1	2	3
Do you think you could get hurt or drown if you move in any way to the surface, without material, in a deep area for a long distance?	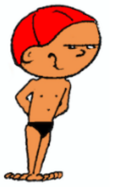 It doesn't hurt / I can't drown	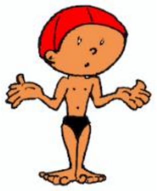 It can hurt me/can drown me	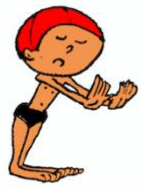 I can almost certainly get hurt/drown.
Do you think it is dangerous to move on the surface, without equipment, in a deep area for a long distance?	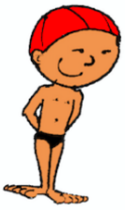 Nothing dangerous	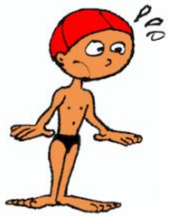 Danger	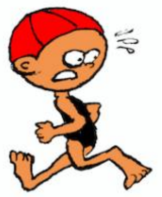 Very dangerous
Would you like to move continuously for a long distance on the surface without the use of equipment?	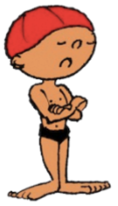 I wouldn't like to	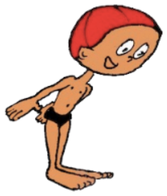 I would like to	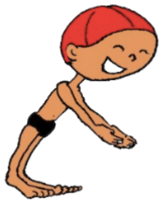 I would really like to
Are you afraid to move continuously for a long distance on the surface without the use of equipment?	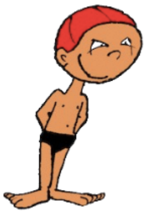 I don't feel fear	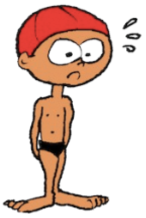 I feel fear	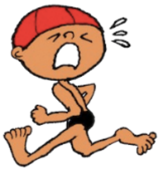 I feel very scared

Motor task

Move in any manner on the surface (front, sideways, backwards) continuously for a distance between 1 and 8 laps (you can change swimming pattern, but you cannot lean on the edge or a float or touch the bottom with your feet), in a deep area, without the aid of equipment.

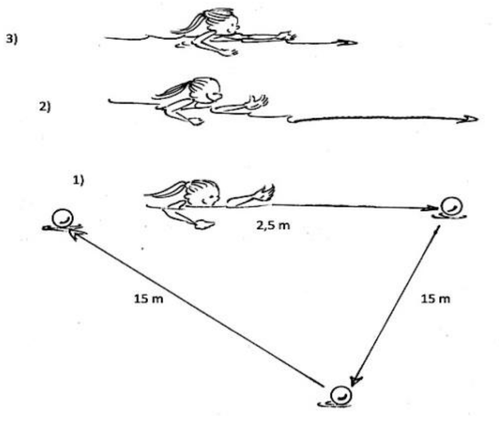

Selected task level		Evaluation criteria			
**What level of more complex task do you think you are able to do very well and want to do?**<br>1) Move between 1-2 laps.<br>2) Move between 3-5 laps.<br>3) Move between 6 and 8 laps.	**How the task is done**	**0**<br>Refuses to do the task or moves for a distance of less than one lap	**1**<br>Needs material support (with or without glasses)	**2**<br>With googgles	**3**<br>Without googles
	**Pauses** (pause for rest during the laps)		**1**<br>2 or more pauses	**2**<br>1 pause	**3**<br>No pauses
	How many swim patterns use		**1**<br>1 swimming pattern	**2**<br>2 swimming patterns	**3**<br>3 or more swimmin patterns

Distance in metros: …………………

Time of execution: ……....minutes ………..seconds

Task 4. Measurement of flotation and aquatic orientation competence.

Questions	1	2	3
Do you think you could get hurt or drown in a situation of floating, while singing a song and changing direction, without the aid of equipment, in a deep area?	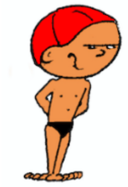 It doesn't hurt / I can't drown	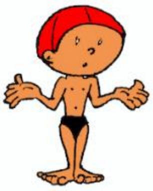 It can hurt me/can drown me	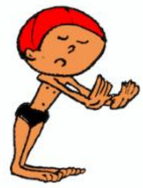 I can almost certainly get hurt/drown.
Do you think it is dangerous to float while singing a song and changing direction without the aid of equipment, in a deep area?	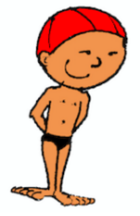 Nothing dangerous	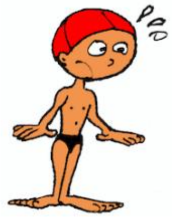 Danger	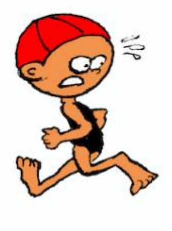 Very dangerous
Would you like to float now, without the help of material while singing a song and change direction at our signal in this deep zone?	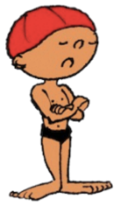 I wouldn't like to	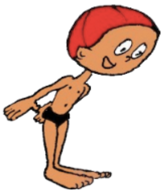 I would like to	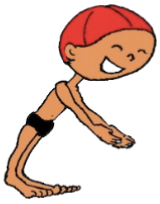 I would really like to
Do you have the fear of floating now while singing a song, without the help of material, in this deep zone and change direction on our signal?	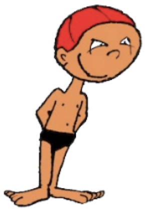 I don't feel fear	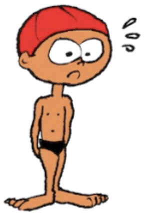 I feel fear	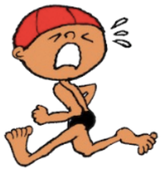 I feel very scared

Motor Task

Float lying on your back in the deep zone for 30'' while trying to sing a song, followed by vertical standing with changes of direction on the educator's signal (with arm actions indicating different directions - forward, right, left, backward - one at a time). Before each change of direction, it is necessary to return to the upright position.

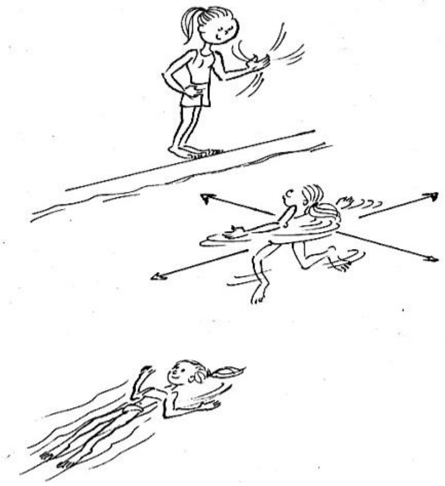

Selected task level		Evaluation criteria			
**What level of more complex task do you think you are able to do very well and want to do?**<br>1) Float lying on your back for max. 30''.<br>2) Flotation lying on your back for max. 30'' + changes of direction with simulated rain.<br>3) Flotation (same as above) + changes of direction with simulated rain.	**How the task is done**	**0**<br>Decline to do the task and his body sinks	**1**<br>Make with help (with or without goggles)	**2**<br>With goggles	**3**<br>No goggles
	**Singing while floats**		**1**<br>Can't sing while floating	**2**<br>Sings with interruptions (swallows water, needs to move to float)	**3**<br>Singing without interruptions
	**Back float (lying on back)**		**1**<br>Difficulty in keeping the body on the surface, many arm and leg movements. Sinking legs	**2**<br>Stays on the surface with slight movements of arms and/or legs. Body close to horizontal	**3**<br>Stays on the surface with body close to or horizontal without arm and/or leg movements
	**Vertical treading while awaiting change of direction**		**1**<br>While waiting for the signal fails to float (body sinks).	**2**<br>While waiting for the signal, floats with the help of fast and disorganised motor actions (crawl kick). Sometimes can submerge the head	**3**<br>While waiting for the signal, float with well performed treading actions (arms in the water, leg sustaining, eggbeater, head out of the water). Always keeping head above water.
	**Directions change**		Can’t change direction	1-2 direction changes	3-4 direction changes

Time of execution: ……….minutes ………..seconds

Task 5. Measurement of recognition competence of a call for help and application of the drowning chain of survival.

**Question**	**1**	**2**	**3**
Do you think you could get hurt or drown if you identify a help request from someone in the water and have to initiate the response because you are the only one present, without having to get into the water to do so?	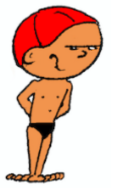 It doesn't hurt / I can't drown	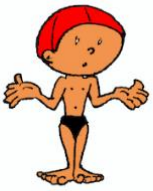 It can hurt me/can drown me	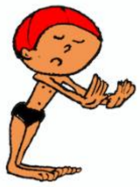 I can almost certainly get hurt/drown.
Do you think it is dangerous to identify a help request from someone in the water and you have to initiate the response because you are the only one present, without having to get into the water to do so?	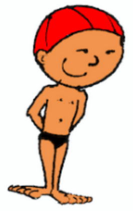 Nothing dangerous	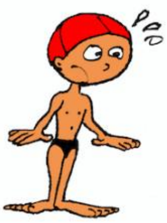 Danger	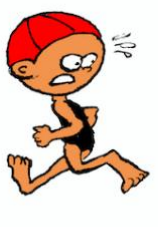 Very dangerous
Would you now like to help a person in the water who is close to you, initiating the response, without getting into the water?	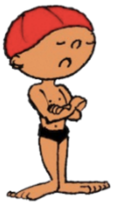 I wouldn't like to	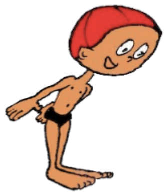 I would like to	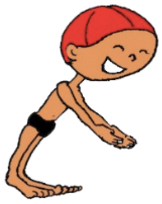 I would really like to
Are you fearful now to help a person in the water who is close to you, initiating the response, without getting into the water because you are the only person present?	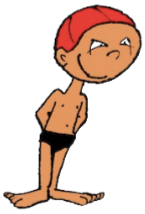 I don't feel fear	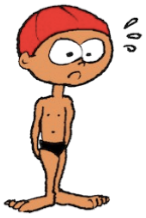 I feel fear	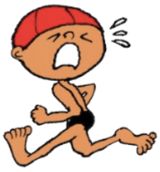 I feel very scared

Motor task

Recognise the emergency request, choose an aid material and provide help from out of the water to a person who is in the water in a deep area. Remove the person from the water and perform first aid (Drowning Chain of Survival).

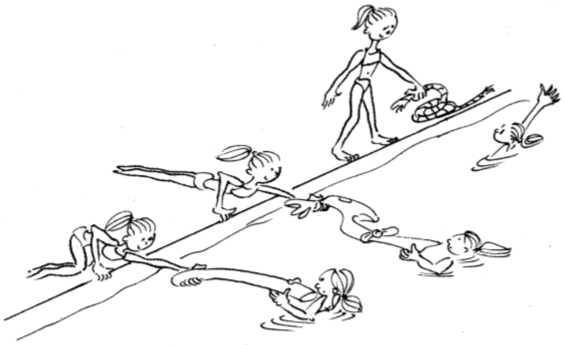

Selected task level	Evaluation criteria				
**What level of more complex task do you think you are able to do very well and want to do?**<br>1) Provide assistance with a tubular float, without going into the water + perform first aid.<br>2) Assist with 1 towel, without entering the water + perform first aid.<br>3) Assist by throwing a rope over a distance of 5 m, without entering the water + perform first aid.	**Assisting** (without going into the water, with auxiliary equipment)	**0**<br>Refuse to do the task or does not know what to do	**1**<br>Uses the aid equipment, but stays close to the person that provides the aid (far away from the injured person).	**2**<br>Use the auxiliary equipment, but it remains far away from the injured person who has to move (maximum distance 1 m / 2 strokes).	**3**<br>Uses the auxiliary equipment and it reaches the injured person.
	**Number of tries**		**1**<br>3 tries and does not reach the person	**2**<br>Reaches the person on the 2nd or 3rd try	**3**<br>Reaches the person with 1 attempt
	**Removing the person from the water**		**1**<br>Forgets to take the person out of the water	**2**<br>Ask for help to get the person out of the water.	**3**<br>Find a safe strategy to get the person out of the water.
	**Life support** (check if the person is conscious, if conscious, put in lateral safety position, if not apply A+B ventilation - 5 breaths + 30 compressions)		**1**<br>Acknowledges the emergency, but remains confused about the steps to be taken	**2**<br>Recognises the emergency and makes a SOS call, but does not initiate A+B ventilation or does it incorrectly	**3**<br>Complete all steps correctly
**Time of execution:**	……….minutes ………..seconds				

Task 6. Measurement of personal flotation device (life jacket) competence.

Questions	1	2	3
Do you think you could get hurt or drown if you have to use a life jacket to move in the water and you don't know how to manage it properly?	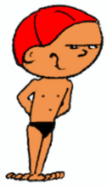<br>It doesn't hurt / I can't drown	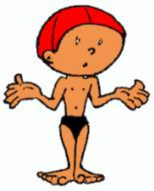<br>It can hurt me/can drown me	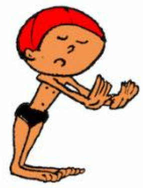<br>I can almost certainly get hurt/drown.
Do you think it is dangerous if you have to use a life jacket to move in the water and you don't know how to manage it properly?	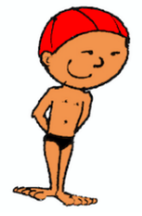<br>Nothing dangerous	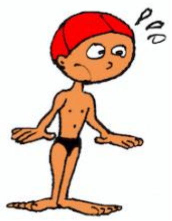<br>Danger	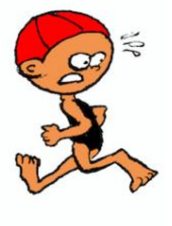<br>Very dangerous
Would you like to use a life jacket to get around in the water now, even though you may not know how to manage it correctly?	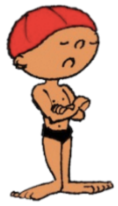<br>I wouldn't like to	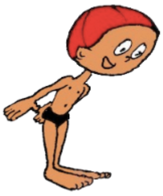<br>I would like to	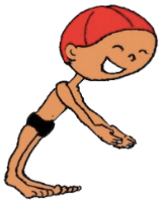<br>I would really like to
Are you afraid to use a life jacket to get around in the water now, even though you may not know how to manage it properly?	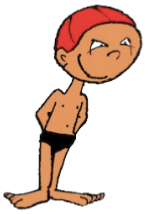<br>I don't feel fear	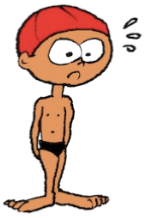<br>I feel fear	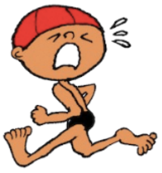<br>I feel very scared

Motor task
Operate and moves for a distance with the Personal Flotation Device (PFD) correctly.

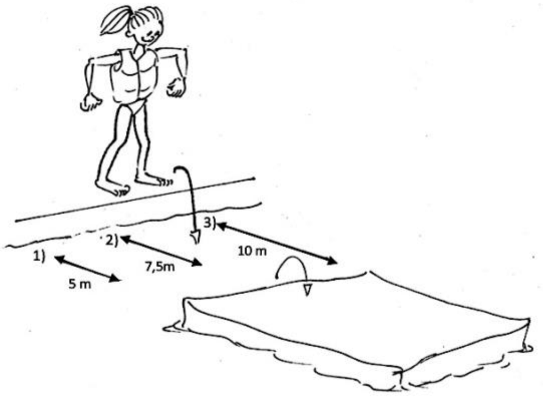

Selected task level	Evaluation criteria				
**What level of more complex task do you think you are able to do very well and want to do?**<br>1) Operate and move over a distance of 5 m and step onto the pool mat.<br>2) Operate and move over a distance of 7.5 m and step onto the pool mat.<br>3) Operate and move for a distance of 10 m and step onto the pool mat.	**Operate life jacket**	**0**<br>Refuses to do the task or fails to move with PFD	**1**<br>Ask for help to manage the PFD	**2**<br>Operates PFD alone, but sets it incorrectly	**3**<br>Operates alone the PFD and adjusts it correctly.
	**Move with life jacket**		**1**<br>Difficult y in moving with the PFD and cannot get on the mat.	**2**<br>It moves easily, but fails to get on the mat.	**3**<br>It moves easily and gets on the mat.
**Time of execution:**	……….minutes ………..seconds				

Task 7. Measurement of 'safe exit' competence.

Questions	1	2	3
Do you think you could get hurt or drown if you fall into the water at a distance of 15m from the exit, in a deep area, have to move towards the exit point and get out of the water on your own?	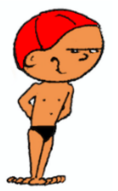 It doesn't hurt / I can't drown	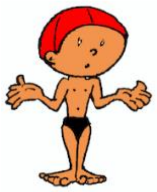 It can hurt me/can drown me	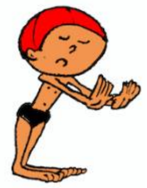 I can almost certainly get hurt/drown.
Do you think it is dangerous if you fall into the water at a distance of 15m from the exit, in a deep area, you have to move towards the exit point and get out of the water on your own?	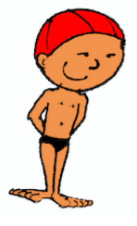 Nothing dangerous	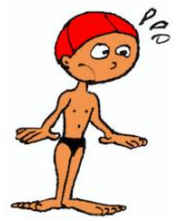 Danger	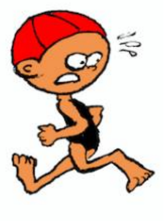 Very dangerous
How would you like to fall now into the water from a floating platform, in this deep area, having to move for 15 metres to the exit point and then get out of the water alone?	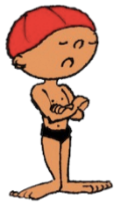 I wouldn't like to	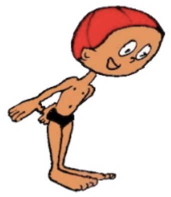 I would like to	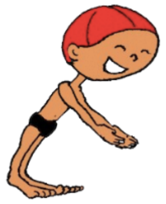 I would really like to
Are you afraid of falling into the water now from this floating platform, in this deep area, having to move for 15 m to the exit point and then getting out of the water alone?	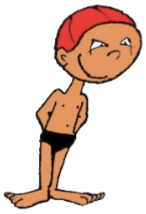 I don't feel afraid	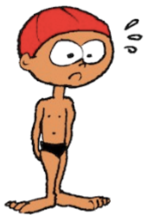 I feel afraid	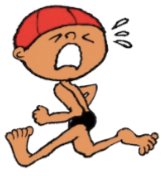 I feel very afraid

Motor task
Find a safe exit after falling into the water from a floating mat (12 cm thick or 2 overlapping mats), in a deep area and move a variable distance to the safe exit point and then exit the water with autonomy.

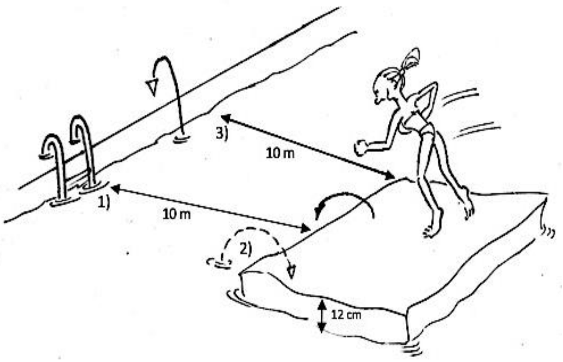

Selected task level	Evaluation criteria				
**What level of more complex task do you think you are able to do very well and want to do?**<br>1) Move for a distance of 10 m and climb up the ladder.<br>2) Jump and return back up to the mat.<br>3) Move for a distance of 10 m and climb to the highest edge of the pool.	**Water exit**	**0**<br>Refuse to do the task	**1**<br>Try but cannot get out of the water	**2**<br>Get out of the water with help	**3**<br>Get out with autonomy
**Time of execution:**	……....minutes	………..seconds			

2.
MEASUREMENT OF THE PREVENTION DIMENSION

Recognition of Local Risks

Read the questions carefully and mark the box with the answer you agree with.

**River Activities**			
1. The type of bottom is an important reference for moving through the water. How do you move on a bottom with rocks and different things (such as seaweed, deeper and shallower areas)?			
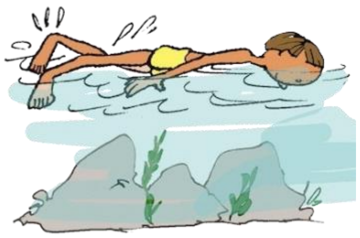		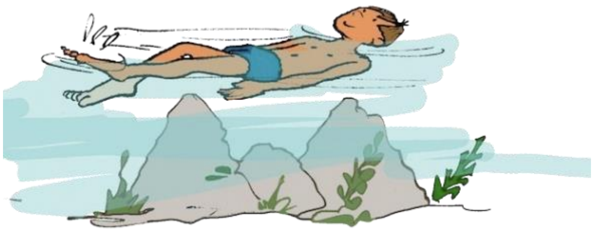	
a. I move face down with my arms at my sides.		b. I move on my back with my arms at my sides.	
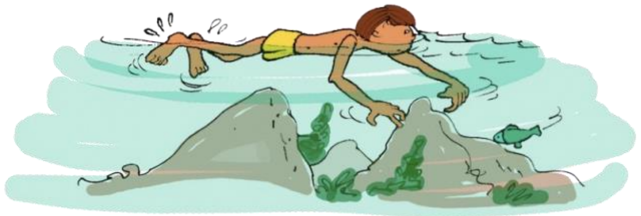		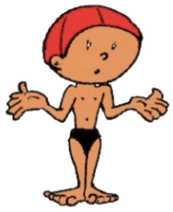	
c. I move with one hand in front of me and my eyes open.		d. I don't know what to do.	

2. Imagine that you are surprised by a current that drags you into a dangerous situation in a river. What would you do in this situation?			
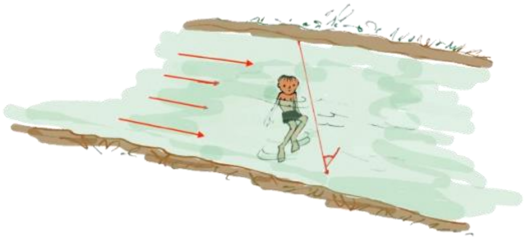		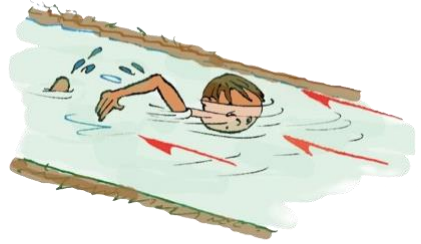	
a. I stay calm, turn on my back, feet first, move diagonally to the river bank and make the signal for help.		b. I lay on face down and move against the current.	
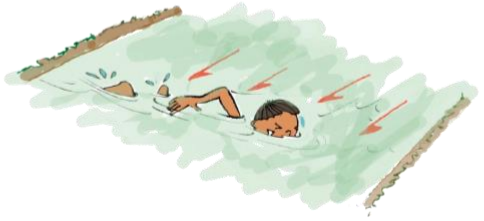		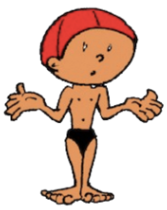	
c. I move perpendicular against the current and appeal for help.		d. I don't know what to do.	

3. The way you enter the water (jumping, etc.) is often the cause of a possible serious injuries. What should you do before entering into the water when you are in a river?			
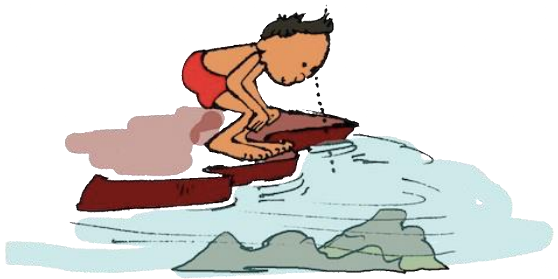		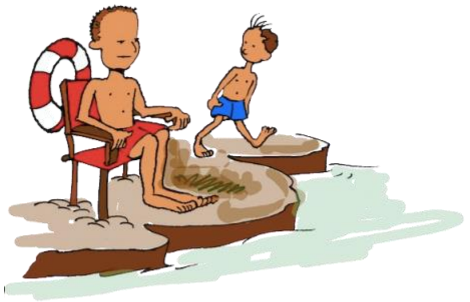	
a. I check that the place is supervised, the depth, the type of bottom, the current and the temperature of the water.		b. I check if there are lifeguards, respect their instructions and the rules of the place by entering the water.	
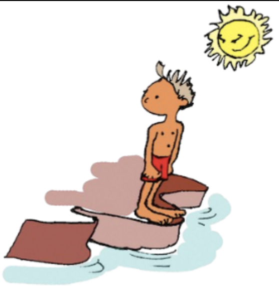		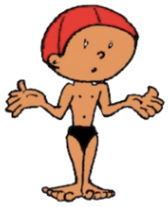	
c. I check that the lifeguard doesn't see me and I enter the way I want to (e.g. head first).		d. I don't know what to do.	

Beach Activities (sea)			
**4. Imagine you are playing in the sea and suddenly a current of water makes you not touch the bottom. What would you do in this situation?**			
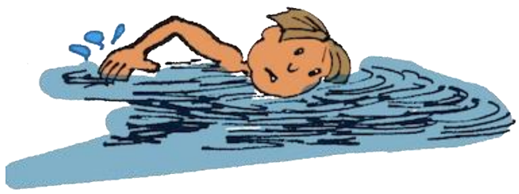		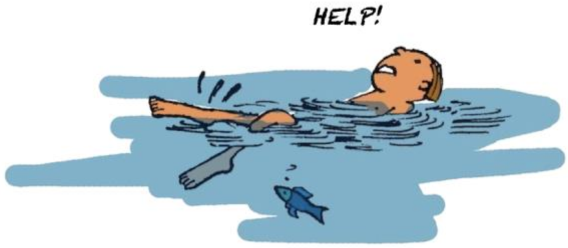	
a. I try to swim against the current.		b. I stay calm, float on my back and call for help.	
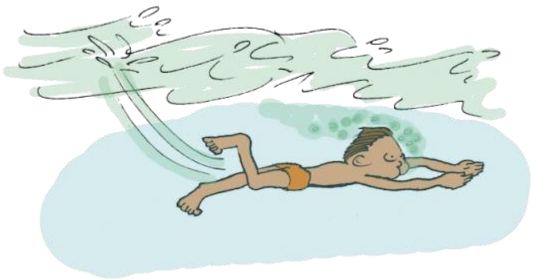		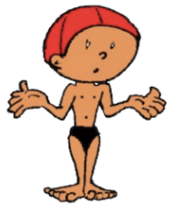	
c. I dive to try to get out of the current.		d. I don't know what to do.	

5. A storm is something that can happen without warning. What would you do if lightning strikes when I'm in the water?			
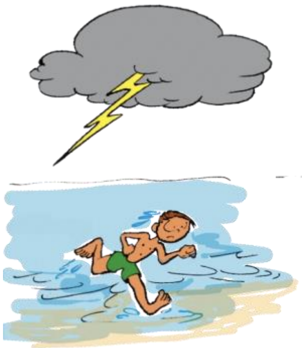		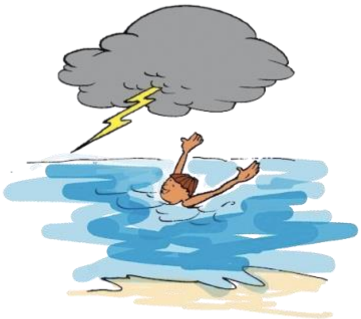	
a. I immediately get out of the water and leave the storm zone.		b. I keep playing in the water because there is no danger.	
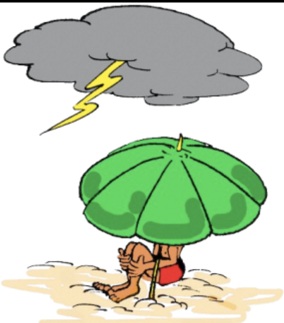		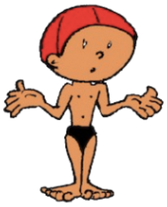	
c. I quickly get out of the water and protect myself under an parasol.		d. I don’t know what to do.	

6. Imagine that you are going to play on the beach with an inflatable device on a sunny and windy day. What would you do if the inflatable device is blown away from the beach because of the wind?			
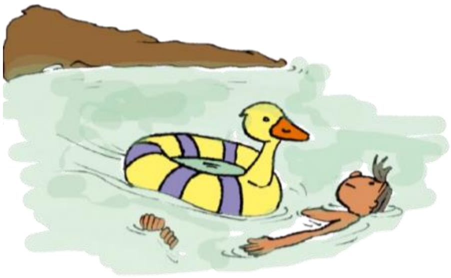		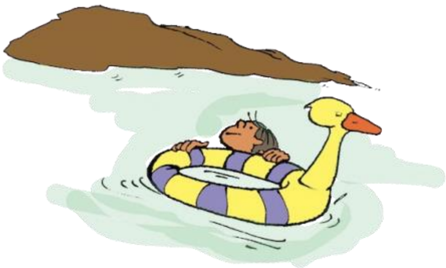	
a. I let go the device, stay afloat and scream for help.		b. I hold the device, ask for help and wait for it.	
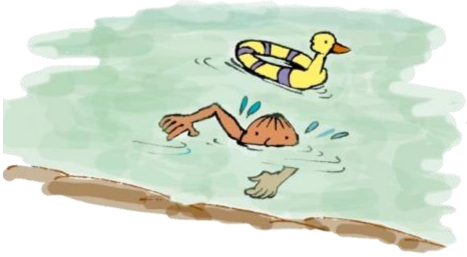		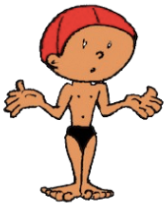	
c. I let go the device and try to swim to the beach, even if I don't know how to swim well.		d. I don’t know what to do.	

Pool Activities			
7. Every aquatic environment has its own unique challenges (every place is different). What should you do when you go swimming/playing in the pool?			
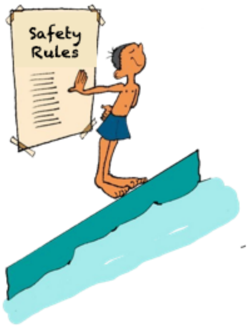		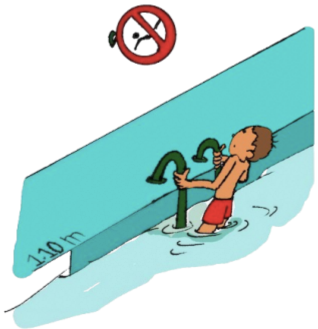	
a. Read the safety rules, but do what I want to do and what I think I am able to do.		b. Read and follow the safety rules for that site or pool.	
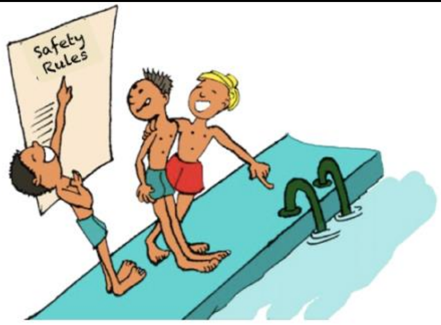		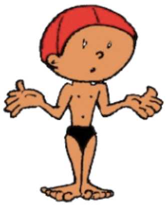	
c. Do the challenges and games with my friends, even if they don't respect the safety rules.		d. I don’t know what to do.	

8. Playing in the pool is very common among children, but there are many dangers. What should you do when you feel tired during a game or can't stand standing in the deep end any more?			
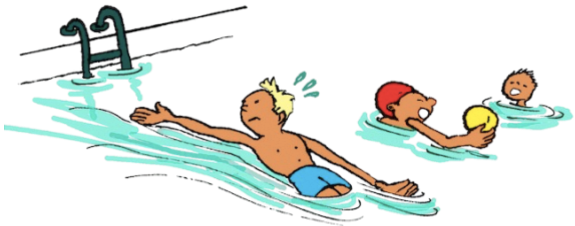		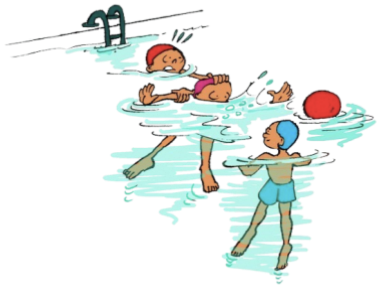	
a. Float to the nearest safety exit (edge, ladder).		b. Hold on to a friend in some way.	
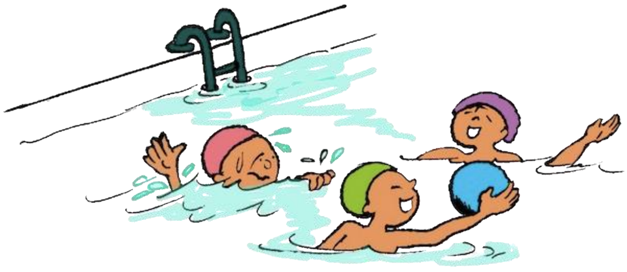		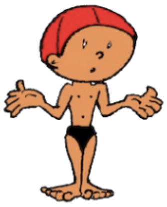	
c. Make quick movements to be seen.		d. I don’t know what to do.	

Activities in the general aquatic environment (sea, river, swimming pool, lake)			
9. When I help a person, I need to ensure my safety and be aware of the risk involved. What is the best way to help a person who is in the water and needs help?			
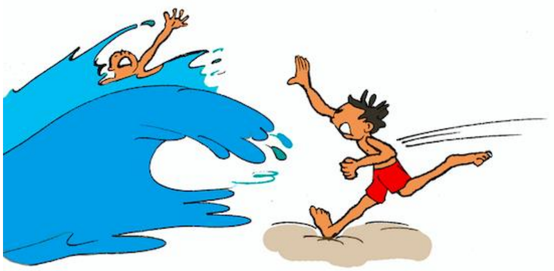		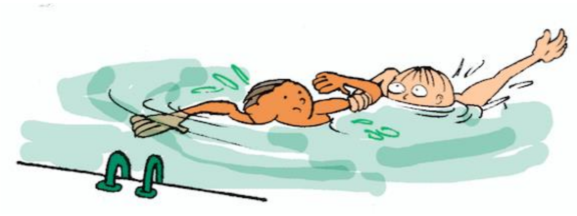	
a. Helping her, even if it's dangerous for me, but her life is important.		b. I go into the water and try to pull it out by myself.	
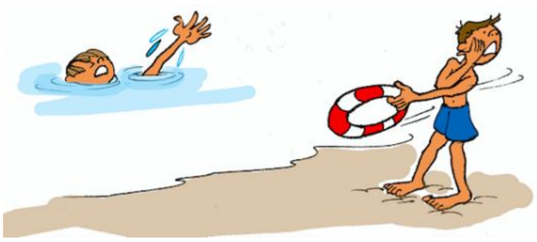		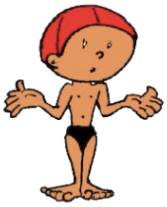	
c. I alert the lifeguard or, if I am alone, I call for help and throw a floating object to assist. Without entering the water or compromising my safety.		d. I don’t know what to do.	

10. Knowing how to float, although very easy and important, is often a big challenge. What would you do to help yourself to float in a dangerous situation?			
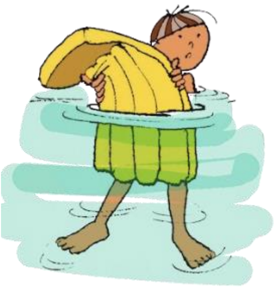		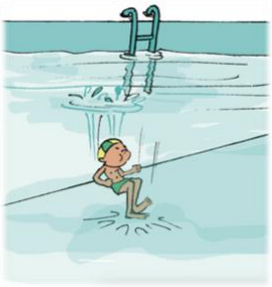	
a. I hold on to a floating material, such as mats, large empty bottles or any other material that allows me to float.		b. I push the bottom to return to the surface.	
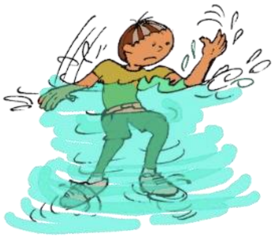		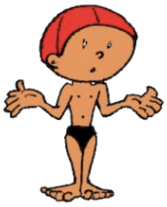	
c. I keep my clothes on and hit the water hard with my arms.		d. I don’t know what to do.	

Signage in Aquatic Environments

1. Match the colour of the flag (letter column) with the meaning of the flags (number column). Follow the example.

a)	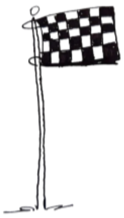 **Chess flag.**	1)	High risk. Very dangerous conditions, such as rough waves and strong currents. Do not enter the water.
b)	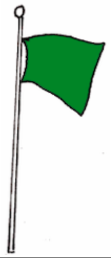 **Green flag**	2)	Beach temporarily unguarded.
c)	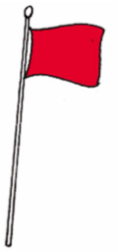 **Red flag**	3)	Medium risk. Extreme caution is recommended during bathing (e.g. no floats, no hip-deep water, no swimming).
d)	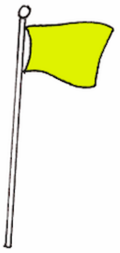 **Yellow flag**	4)	Low risk. Good conditions for bathing and swimming, in accordance with safety recommendations.

You can answer here: a) 2 b) c) d)

2.	**Match the figures (letter column) to their meaning (number column).**

a)

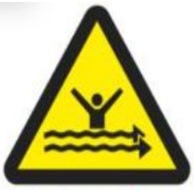

b)

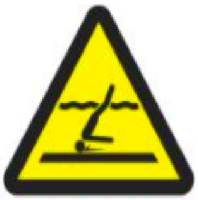

c)

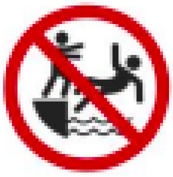

d)

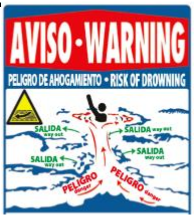

1) Danger and risk sign in swimming pool. Shallow water.

2) Prohibition sign in swimming pool. No pushing into the water.

3) Signs of dangers and risks on the beach. Area with strong currents, consult the lifeguard before entering the water.

4) Danger and risk sign. Return current, consult the lifeguard before entering the water.

You can answer here: a) b) c) d)

Wind, wave and tide conditions

3.	Match the figures (letter column) to their meaning (number column).

a)	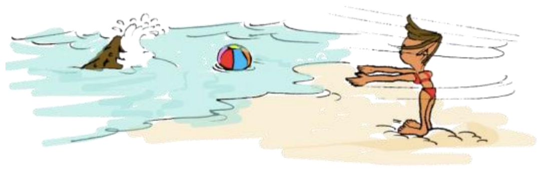<br>**A strong wind blowing from land to sea.**	1)	I should not go into the sea because of the danger of this type of waves that indicate a storm.
b)	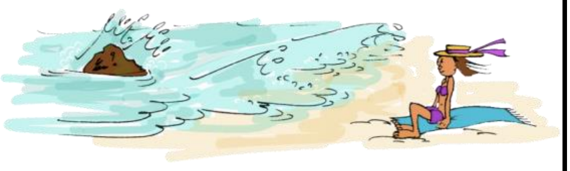<br>**Disorderly, irregular waves of variable size.**	2)	It indicates the strength of the water, its ability to push towards land and the possible slope of the beach, so you should consult a lifeguard or avoid swimming.
c)	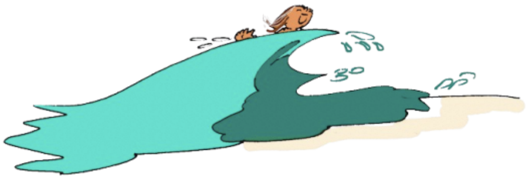<br>**Waves and size of the foam.**	3)	I must not enter the sea with a floating material, nor swim towards it, as it will blow me out to sea with the force of the wind.

You can answer here: a) b) c)

In this series of questions, mark with an X the answer (only one) from 1 to 4 that best represents your choices in these situations. There are no right or wrong answers, it is very important that you answer honestly.

Respect between equals					
**Questions**		**1 (Never)**	**2 (Rarely)**	**3 (Frequently)**	**4 (Always)**
**1**	Have you ever been pressured by one or more friends to get in the water and do something you shouldn't or didn't want to do?				
**2**	Do you think that if you don't accept the aquatic challenges presented to you by your friends you will lose the respect of the group and/or you will no longer be accepted?				
**3**	Do I find it difficult to say no to my friends even when I know the situation is dangerous (e.g. jumping from a high altitude or from a bridge)?				
**4**	Sometimes I do dangerous things (do the somersault into the water) to attract the attention of others (impress)?				

In this set of questions, mark with an X the answer (only one) from 1 to 4 that best represents your choices in these situations. There are no right or wrong answers, it is very important that you answer honestly.

Cognition about level of risk in risky situations					
**Questions**		**1 (Without risk)**	**2 (Low risk)**	**3 (High risk)**	**4 (Extreme risk)**
**1**	Imagine you see your colleague drowning. Do you think you would be ready and willing to help him or her without putting your life at risk?				
**2**	In your opinion, do you think it is risky to perform an acrobatic jump (somersault) to enter the water?				
**3**	In your opinion, do you think it is risky for your partner to encourage him/her to do something in the water that he/she does not want to do (go in the water if he/she does not know how to swim or feels cold)?				
**4**	In your opinion, do you think it is risky to go on a boat (boat, kayak, paddle, etc.) and not wear a life jacket?				
**5**	In your opinion, do you think it is risky to help a drowning person without being trained in water rescue?				
**6**	In your opinion, do you think it is risky for your partner to be pushed into the water?				

Always start sentences with 'I believe that.....' and choose the answer from 1 to 4 that best represents your way of thinking. There are no right or wrong answers, your truth is paramount.

Beliefs					
	**Questions**<br><br>**"i believe that..."**	**1 (Strongly disagree)**	**2 (Slightly disagree)**	**3 (I somewhat agree)**	**4 (Totally agree)**
**1**	'Getting injured or drowning in a swimming pool only happens to other people'.				
**2**	'Sometimes children get injured when they are in the pool, river, lake or beach, but it's not usually serious.				
**3**	'I can play in the sea with peace of mind, because I know how to swim in the pool'.				
**4**	'If I get injured in the river it's because of bad luck and not because I did something wrong'.				
**5**	'If I can swim, it is less important to wear a life jacket on a boat, as there is no risk of injury or drowning.				

3.
MEASUREMENT OF THE ENVIRONMENTAL EDUCATION DIMENSION

Answer each question by choosing the coloured face that best represents what you like to do or observe in the different situations presented.

Red - means you don't like it**; Yellow -** means you like it more or less and **Green -** means you like it**.**

**How much do you like to observe or do the following things in a natural aquatic environment (beach, river, etc.)?**		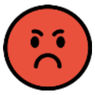	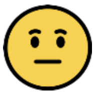	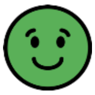
**1**	Observe and get to know the algae that live in the marine ecosystem in which I swim or visit (in their natural environment).			
**2**	Picking up trash and food scraps that I generate and/or find around me.			
**3**	It is important to know the differences between a domestic animal and a wild animal. I am curious to know and preserve wild animals (crabs, snails, limpets, mussels, clams, etc.).			
**4**	Enjoy spending free time in natural aquatic environments.			
**5**	Learn more about aquatic environments (characteristics, preservation, richness, etc.).			
**6**	Playing with small stones by the seashore/broken shells on the beach.			
**7**	To be part (myself) of nature, trying to interfere as little as possible with the ecosystem (e.g. not to handle animals, shells and stones from the site).			
